# Advice to early career nutritionists on working in and with the food industry

**DOI:** 10.1111/nbu.12730

**Published:** 2025-01-12

**Authors:** David J. Mela

**Affiliations:** ^1^ Valkenswaard The Netherlands

**Keywords:** collaboration, integrity, publications, research

## Abstract

Early career researchers (ECRs) in nutrition and related fields often wish to approach commercial organisations for possible funding or collaboration in scientific projects and other activities. However, ECRs may experience challenges from their limited experience, lack of understanding of the food industry and concerns about working practices and research integrity. This commentary is oriented toward providing some basic, practical guidance for nutritionist scientists, to help in developing credible, principled and effective working relationships with the food industry. Based on the author's experience as an academic and industry researcher, and an advisor to academic‐industry collaborative projects, the text addresses a range of related aspects including: understanding and approaching the food industry; the industry environment and drivers; contracts, confidentiality and communication; potential challenges; and ensuring scientific integrity.

## INTRODUCTION

Students and early career researchers (ECRs) working on topics related to food and nutrition may often have contact with the food industry, through professional meetings, collaborative projects or perhaps as potential employees. They may also seek industry contacts to discuss new ideas, potential collaborations or contributions. Working with industry gives access to additional expertise, capabilities and facilities, and potentially interesting and meaningful (impactful) research questions. For many academics, however, the ‘food industry’ is something of a mystery. It also comes with a dubious reputation for some of its products and practices, raising questions and concerns around ethics, trust and integrity. While many public funding streams encourage or even require partnerships with industry, there is also a widespread uneasiness about the role of industry in nutrition research, and the conflicts of interest that may emerge from such relationships.

The intent of this piece is to give some honest and practical insights into the nature of the food industry and advice on working practices and collaborations. It also considers some issues that may arise, and warning signs and pointers to promote professional relationships and research integrity. It is written from the perspective of someone who has seen the good, the bad and the ugly, both as an academic and food industry research scientist.

## REACHING OUT: THE ‘FOOD INDUSTRY’ IS NOT A SINGLE ENTITY

The food industry ranges from raw material production (e.g. farmers) through food and ingredient manufacturers, to consumer point of purchase (retailers and food service). It also encompasses a broad range of trade organisations and supporting services (research, regulatory, analytical, marketing, etc.). These commercial entities have widely differing sizes, interests, business models, objectives and challenges.

A company's ‘portfolio’ describes the range of raw materials and products their business is based around, and the major brands and markets where they appear. This is important to know, both in seeking the right potential research partner and having an informed discussion with them. Some companies rely heavily on a single commodity source (soy, cocoa beans, tea leaves, chicken…) whereas others, such as large manufacturers and retailers, use an extensive range of raw materials. Even a single commodity can, however, be the basis of a wide range of differing products and markets (e.g. dairy ➔ milks, yogurts, ice creams, protein isolates, meat replacers, etc.). But there are also companies whose business is really based around a single product, perhaps a food supplement or speciality ingredient like a specific sweetener or isolated fibre. The business models may also vary widely; for example, products may be sold mainly business‐to‐business as component ingredients, or as retail products under brands that target different outlets and consumer segments.

Companies also vary widely in their internal research and development (R&D) capabilities. Large multi‐national food producers might have hundreds of PhD‐level scientists with research experience, plus other related R&D support. They publish and present research papers and engage actively with an international academic network. In contrast, small companies, those with a narrow portfolio or a focus on retailing may have only a few science experts and limited internal R&D facilities, so buy‐in services and expertise as needed. The level of internal R&D staffing and capabilities will have a significant impact on the nature of any scientific discussions and collaborations, and what the company may expect from them. Large manufacturers will look to academics for more fundamental understanding that extends their internal capabilities, while smaller companies and retailers may seek more ‘close to market’, readily translatable research.

This background information may be helpful in thinking about the most relevant and promising industry partners for a particular piece of research. But it can still be challenging, especially for ECRs, to identify and approach potential industry contacts. An easy first step is to speak with colleagues and more experienced researchers who have worked with industry about their own networks. They may have commercial contacts who could be relevant or offer advice based on their knowledge of other companies. It is also useful to search specifically for companies with a history of participation in national or international research programmes, or industry scientists with a record of publication or presentations in your topic area. Those individuals who already share a professional knowledge and interest in your field can be an excellent and particularly approachable starting point.

**Table:** 

**Before approaching industry, do your homework**.
Learn about the companies you are approaching—their brands and products (‘portfolio’), existing research capabilities and likely challenges.
Then be prepared to listen to their interests before sharing yours.

## ENGAGING: DIFFERENCES BETWEEN ACADEMIC AND INDUSTRY ENVIRONMENTS

In developing a collaborative relationship or considering a position with industry, it is useful to be aware of how the working environment and mindset differs from an academic research setting. Some of these differences are captured in Table 1.

**TABLE 1:** Differences between the academic and industrial research mindset.

Academic drivers:	Industry drivers:
Scientific curiosity (‘What if…?’)	Translational science (‘So what?’)
Professional advancement (publications, impact)	Commercial direction and advantage (innovation, cost, sourcing, know‐how, claims)
Individual expertise	Network of expertise
‘More research is needed’ (uncertainty good for business)	Decisiveness, ‘go/no‐go’ (consensus good for business)

### Internal and external drivers

Academics are largely driven by their personal research interests and curiosity, although this is significantly channelled by the external environment (ability to generate funding, publications and impact). In contrast, industrial science is largely driven by the external environment, undertaking product innovation and renovation to address specific market or regulatory challenges and to gain economic and competitive advantages through cost savings and improved product features.

Companies are not charities. They ultimately need to make a profit to stay in business. In some areas there is tremendous price competition and the earnings (margins) on sales are tiny (e.g. supermarkets), while in other areas the margins are huge (e.g. supplements). In either case, they are rarely giving away their time, expertise and money without knowing ‘What's in it for us?’. When discussing collaborative projects, it is therefore valuable to ask about and understand these challenges and potential opportunities, and be clear where a proposed activity realistically fits with these. Be prepared to succinctly say how a collaboration based on your idea could change how the company operates, what the tangible impact might be. In short: what could this look like if successful?

### Competitive advantage and intellectual property

Academics, especially ECRs, usually want to communicate their latest research ideas and results as quickly and widely as possible. It is done with pride, and encouraged by institutions and granting agencies. And academics also need to position themselves favourably against their scientific colleagues for funding, recognition and career advancement.

In contrast, companies may sometimes adopt a more cautious approach to communicating research outputs, in order to benefit from early access to new knowledge or technologies. They are seeking to capture a potential ‘competitive advantage’, which may be derived from the ability to source, manufacture or sell products or services with new or improved functionality or quality, better margins (lower development, material or production costs) and greater perceived consumer value—preferably in ways that are difficult for competitors to match. This can be achieved in many ways, such as favourable access to materials, facilities or distribution channels, or intellectual property (IP) in the form of proprietary production or technical capabilities.

Importantly, IP is not limited to legal rights of use such as patents and licensing. It also includes ‘trade secrets’ and technical expertise that may be carefully guarded. The other important side of IP is ‘freedom of operation’, establishing that there are no barriers to adopting a new process or technology—particularly whether it might infringe on existing patents. You may not be able to patent your idea, but you also want to be very sure no one else already ‘owns’ it!

### Contracts and confidentiality

For any collaborations with industry, it is essential for academics to get early guidance from their legal or contracts office. Most granting agencies have standard contractual arrangements for research with industry using public funding, and it is helpful to understand these general conditions before seeking industry partners. For research directly funded by companies, it is common for the industry partner to draft a contract, which is then agreed with the academic research site through negotiation. Unfortunately, this can sometimes be a protracted and painful process, underscoring the importance of establishing the right internal contacts and understanding the general policies operating at your site.

‘Confidential information’ is usually legally defined in non‐disclosure agreements (NDA), which may be required even to begin a discussion of a potential research collaboration. In practice, anything that could not be learned through publicly available sources should be treated as confidential. This will often cover proprietary information companies want to share about their products, technology or marketing. Academics should however be very cautious about signing broad confidentiality agreements covering quite general topic headings. This can create a risk of so‐called ‘information contamination’, if it covers knowledge that you might gain or pursue anyway through your own, other independent routes. A confidentiality agreement also should never prevent you from publicly disclosing the existence of a working relationship with a company, information which is needed for, for example conflict of interest declarations.

**Table:** 

**Caution on contracts and confidentiality**
Engage early with your legal/contracts office to understand the basic conditions and processes that may apply.
Do not exchange confidential information or sign confidentiality agreements unless and until it is really necessary.
The scope of ‘confidential information’ should be defined as clearly and narrowly as possible.
Be discreet and respectful of any knowledge you gain about companies you work with, even if it is not formally covered by an NDA.

### Risk‐taking: ‘Fail early, fail cheap’

Academic research is typically supported by publicly funded, multi‐year grants, allowing investigators to test and build upon their favoured hypotheses. These arrangements provide for considerable intellectual freedom. However, this system of funding may also encourage academics to keep even bad ideas alive and funded as long as possible and avoid defining any truly decisive (potentially idea‐killing) ‘go/no‐go’ experiments or milestones. This same mindset may also be found in some smaller and start‐up companies, heavily dependent on the success of a narrow product range. In contrast, larger companies with broader portfolios are motivated to kill off bad ideas as early as possible, with a minimum of investment, allowing resources to be shifted toward more promising alternatives. A decisive ‘failure’ from good research can therefore be seen as a success if it happens early and cheaply.

Only a very limited range of innovative ideas in nutrition and health ever make it to the marketplace. Academics in nutrition and health mainly focus on efficacy, whether an intervention or exposure has beneficial effects on (or associations with) desired outcomes. Companies are certainly concerned with this as well, but also much more (Table 2). It is not difficult to understand the high risk of failure if you take account of all these factors. For ‘functional’ ingredients, especially those based on novel materials, the starting probability of success may be considerably less than 5%. Those odds may still be acceptable, depending on the investment required and potential market value. It makes sense though for companies to decisively confront the most likely hurdles (the biggest potential ‘show‐stoppers’) early on. Thus, fail early, fail cheap.

**TABLE 2:** Feasibility and marketing issues for ‘healthy’ and ‘functional’ foods and ingredients.

Substantiation of beneficial effect, plus:
Regulatory/safety approval
Sourcing route (reliability, quality, sustainability)
Sensory acceptability
Stability to processing (e.g. temperature, shear)
Distribution channel (e.g. ambient / chilled / frozen)
Shelf life
Scale‐up potential and capital requirements
Cost implications
Lifecycle environmental impact
Freedom of operation and proprietary opportunities
Additional for ‘functional’ ingredients:
Suitable food vehicle (technical, legal, ethical)
Permitted, attractive claim
Conditions (dose, frequency) for beneficial effect/claims
Analytical specification of functional entity

### Teamwork

Academics traditionally work on projects headed by a senior investigator and carried out by individuals or small teams with largely similar expertise. There may be a sense of competition with other groups in the same university or perhaps even the same department. That kind of ‘silo mentality’ is strongly rejected in corporate R&D, where reliance on cross‐function teams is the norm. The projects in larger companies are often complex, with input from diverse functions needed not just to do the research but also to assess and translate it into market impact. Close and coordinated teamwork is expected amongst groups with quite different scientific disciplines, as well as technical production, regulatory, marketing and other functions. Corporate R&D works best when it makes full use of the range of dedicated expertise, rather than a few individuals trying to do it all on their own.

## ACADEMIC‐INDUSTRY RESEARCH COLLABORATIONS: ‘NICE TO DO’ VERSUS ‘NEED TO DO’

**FIGURE 1:**
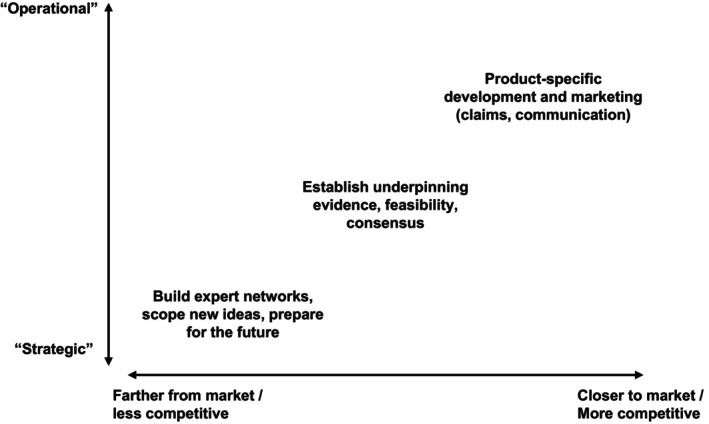
A typical industry perspective on the positioning and purpose of different types of academic research collaborations.

There are a variety of arrangements through which companies work with academics, differing in terms of purpose, priority, sensitivity and timelines. From the industry perspective, research broadly falls along a continuum from ‘strategic’ to ‘operational’, roughly corresponding to their distance from real products or market applications. These are illustrated in Figure 1.

Strategic research (‘nice to do’) is intended to build a general understanding and expert networks around a topic area or idea and scope the potential for further development or applications. This is often exploring emerging areas or addressing areas of interest broadly shared across an industry sector (thus ‘pre‐’ or ‘non‐competitive’). Timeframes can be relatively long, the organisational and financial commitments low, and results (‘success’ or ‘failure’) have limited immediate business impact. In contrast, *operational research* (‘need to do’) is more closely aligned to company projects and plans, and specific product lines and business goals. These activities have greater immediacy and priority. They are usually more company‐specific and commercially sensitive, with results having more direct and important business ramifications.

### Public–private partnerships (PPP, consortia)

PPP are usually pre‐competitive, shared strategic research with multiple academic or non‐academic (industry or non‐governmental organisations) partners. The work itself is often driven by academics, with varying degrees of steering or support (products, facilities, expertise) from companies. There is likely to be limited potential for generating commercially sensitive intellectual property (know‐how or patents), and correspondingly low hurdles to open dissemination of the project and results.

### Sponsored research grants

These are typically research grants to a single research group, from an individual company with a strong interest in developing a specific topic area, expertise or application. Usually more strategic than operational, the specific activities within the research area may be rather loosely defined. This kind of arrangement is mainly of interest where companies already have substantial internal scientific capabilities and want to expand these by having a close relationship with an expert academic group. Compared to contract research (below) the academic group will have greater latitude in determining how the funds are used. The company is likely to monitor the work for potential impact and intellectual property and may impose limited conditions (possible time delays for clearance) on public disclosures.

### Contract research

This is usually a specific piece of operational research, intended to fulfil a work plan or objective largely defined by the company, using advice, services or facilities of an academic team. While the research question may have a narrow focus, academic partners may have opportunities to add elements that increase the scientific interest and novelty. There is likely to be some degree of confidentiality, but academics should still ensure that the protocols are pre‐registered, and research ultimately published without undue restrictions or interference.

### Consultancy

Consultancies can be wide‐ranging, including participation in advisory panels or strategic discussions, input to the design of tests or trials, sponsored presentations for internal or external audiences, or preparation of research papers and literature reviews. These often focus on specific scientific or regulatory challenges or uncertainties, where the industry partner(s) needs independent, authoritative opinions and advice. Discussions and reports within companies are usually confidential, but academics should always have the freedom to publish the results of literature reviews or other assessments based on existing published data.

Consultancies are usually arranged with an individual acting either as salaried staff contracted out by their employer (university) or as an independent contractor. The former route can sometimes be complex, with contracts and costings arranged by the employer, and fees unlikely to go directly to the individual. Academics should be clear on their organisation's rules for this kind of work. For consultancy as an independent agent, individuals will usually need to establish themselves as a verifiable, registered business. The fees paid to independent scientific consultants depend on their stature and qualifications, and the nature of the work. In addition, it is reasonable to charge a modest ‘start‐up’ fee for time spent on the initial discussions and contractual and financial arrangements.

**Table:** 

**Consulting: Know who will use your work and how**
There is an important difference between support for R&D (science) and support for marketing (promotion)!
Be cautious about appearing to represent or endorse a company or its products versus helping companies understand and communicate a factual and balanced view of scientific evidence or issues.

## CHALLENGES

### Managing relationships

Developing and managing relationships with industry partners can sometimes be difficult. Industry contacts are likely to have multiple roles, projects and contacts, with considerable demand on their time and attention. Commercial organisations tend to have hierarchical structures that emphasise delivering on priorities set by senior management. Unfortunately, your project will not always be the highest priority in a busy schedule. Your contact's availability and priorities can also shift rapidly if there is a need to respond to newly‐arising issues.

**Table:** 

**Communication is key:**
Do not rely on e‐mail and messaging. Aim for regular personal contact, also visiting the company in‐person if possible. Fix dates for brief periodic discussions and updates.
Be pro‐active and honest informing about progress, changes or delays.
Identify and communicate early about any possible intellectual property, publications or presentations.

### Organisational complexity and decision‐making

Larger food companies often have multiple, complex organisational structures. Major corporate divisions may be stratified along lines of national or regional geography, product types or distribution channels, brands and corporate functions (finance, marketing, R&D, etc). This makes it difficult for academics to identify the best entry points and individuals within those companies to engage with. Complexities in contracts, budgeting and approvals can be very frustrating (also for people within the companies). This all leads to one of the odd paradoxes, that ease of getting research funding may be inversely related to the financial resources of a company (more money ➔ more difficult to get at it).

An important division for academics to recognise is that between R&D and marketing. Marketing contacts will have a good overview of the business challenges and goals, the customer base, and opportunities they foresee for new consumer benefits and market growth. Some marketeers may also have a good grasp of product technology. However, they may have a limited background in science and research, and that can give rise to unrealistic expectations. In general, academics will find it easier to approach and have meaningful discussions with their industrial R&D counterparts. On the other hand, in the author's experience a convincing letter to the company CEO can sometimes get the desired attention(!).

### Re‐organisations, sell‐offs, takeovers, mergers and acquisitions

A reality of working with and in companies is the potential for very rapid change, due to shifting business priorities. A collaboration aimed toward a particular product type (e.g. bread or ice cream) is not of much interest anymore if the company stops making or sells off that product range. Or maybe that product division is re‐organised, adopts a new marketing strategy or is taken over by a larger division or another company. The original aims of the collaborative project may no longer be of interest, or the key company contacts re‐assigned elsewhere. This can be particularly problematic where researchers are anticipating substantial in‐kind industry support (expertise, facilities or products).

When this happens, companies are still obligated to fulfil contracted commitments. However, managing the relationship can be difficult and frustrating for academics without a clear corporate priority or contacts. While there is no way to prevent such situations, there are ways to reduce the impact. In particular, close and regular communication with corporate contacts can potentially help to anticipate and manage changes, to minimise disruption and ensure some degree of continuity.

### Publications and publicity: Good news and bad news

In almost all cases, academics should have the right to publicly declare the existence and nature of any commercial relationships and to publish and present the results of collaborative research without undue restrictions. The International Committee of Medical Journal Editors (ICMJE, 2024) advises that ‘Authors should avoid entering into agreements with study sponsors, both for‐profit and non‐profit, that interfere with authors' access to all of the study's data or that interfere with their ability to analyze and interpret the data and to prepare and publish manuscripts independently when and where they choose’. The ICMJE and many journals also set out specific criteria for authorship, which can be a valuable reference for deciding which individual academic or industry contributors would be included (or not) as co‐authors.

To respect the working relationships, academics should give industry contacts early indications of intended publications or presentations (what, where, when), and apply caution in any messaging through non‐professional (e.g. [social] media) channels. As has been noted, there may be valid reasons for a delay in dissemination to allow for possible patent filings or ensure communications do not reveal company confidential information. It is also understandable that companies will have an interest in how communications refer to their specific products or marketing. However, the duration and purpose of any corporate delay or clearance procedure should be clear—it is not for redacting or adding ‘spin’ to the manuscript(!). ‘Clearance’ is not the same as ‘approval’.

In the case of ‘good’ news, academics should be explicit about the limits of what can be said or concluded, and avoid appearing to promote or endorse specific companies or products. Unfortunately, commercial organisations (and universities as well) can be paranoid about perceived ‘bad news’. Relationships that seem open and friendly can turn cold when academic research links a food material, process or marketing to potentially adverse effects (on health, the environment, etc), or find that a promising ‘functional food’ or technology does not deliver as hoped. Larger companies have valuable corporate and brand equity to protect, and their communications offices may seek to influence the messaging. Small companies may be surviving on a knife‐edge of profitability or dependent on positive stories to ensure the continued flow of funds from optimistic investors. There may be new, post‐hoc questions about the research design, analyses or conclusions. These are unlikely scenarios, where academics benefit from having followed recommended guidance on scientific integrity (below) and clarity on rights to publish.

On the other hand, there are also reasons why companies may not just acknowledge ‘negative’ outcomes, but actively encourage their publication. If the current scientific evidence is changing, companies benefit from knowing this and promoting change across the industry (vs. acting alone). There is also an industry‐wide benefit to transparently identifying potential safety issues or challenges to communication and claims. And if it is clear that a promising ‘functional’ ingredient or technology does not work, publication ensures a level playing field (prevents‐ others from making false claims).

Regardless of whether it is good or bad news, academics should stick firmly to the facts and the agreed timings and routes of external communication (presentations or publications) and public data sharing. The best advice is also to limit communication of research results to a fair and neutral statement of the evidence and avoid being drawn into undue speculation about the implications (good or bad) for specific companies or products. Although it might be tempting, that is not the role of academics and heightens concerns around the nature of the relationship and conflicts of interest.

**Table:** 

**Stay neutral and fact‐based:**
Your results may be used to re‐formulate or develop new products, to support commercial communication or claims, or change how products are marketed. It may stimulate or kill off a potential line of innovation.
Be sure you can stand behind the research and are clear what it says (and does *not* say). Do not go beyond a neutral statement of the facts.
If others may also communicate your results, agree a clear ‘Can say/Can't say’ document. This can be largely drafted before the research is carried out, based on different outcome scenarios and how they would be interpreted.

## ENSURING SCIENTIFIC INTEGRITY

Working in and with industry raises concerns about a loss of perceived credibility and independence and possible accusations of bias and conflict of interest. There is a wide range of views on the nature and desirability of academic‐industry collaborations in nutrition, as well as the risk from conflicts of interest (Mela, 2024; Mozaffarian, 2017; Soares et al., 2019; Tempels et al., 2017; WHO European Region, 2024). These issues are not straightforward, reflecting differing perspectives within the expert community and funding bodies and the complexity and diversity of interests across the commercial food industry. On the one hand, companies may invest enormous resources into efforts to improve the nutritional values and sustainability of products (e.g. through raw materials and re‐formulation), and understanding consumer drivers for these benefits. Much of the public funding for academic‐industry collaboration in nutrition is intended to deliver toward these well‐intentioned goals. Yet the same companies may also sell products or engage in marketing practices that are seen as detrimental to public health. Some academics may therefore choose not to work with the food industry (or specific companies) at all, and those that do risk the implication that they are complicit with a dubious corporate agenda (Cullerton et al., 2024; Legg et al., 2021; Mialon et al., 2018). It has been suggested that this risk can be managed by keeping industry partners at a distance, perhaps minimising or excluding any role in framing or designing the research (e.g. Cullerton et al., 2024). However, that approach may not be desirable or compatible with research programmes and collaborations intended to address commercially relevant issues using a synergy of expertise.

In practice, the strongest defences against these concerns are to be clear about the purposes and potential use of the research and roles of the partners, and to maximise the transparency, quality and rigour of the processes and working relationships, leaving little room for criticism of the integrity and intent of the collaboration. There are many sources of guidance on working practices and research integrity in general and specifically considering academic‐industry collaborations (Kretser et al., 2019; Kroeger et al., 2018; Larrick et al., 2022; Petersen et al., 2021; Rowe et al., 2009; Schwab et al., 2022; Vorland et al., 2021). The main advice is not rocket science: it largely boils down to having clear and transparent working relationships and scientific objectives, a well‐disciplined approach to the research design and statistical analyses, and making these public in detail (via registries and published protocols) prior to undertaking the research. In exceptional cases, where it is important to keep the research confidential prior to publication (e.g. to protect patent opportunities), it is possible to pre‐register research with an embargo, whereby the registration text is only made public at a later date (such as via the Open Science Framework; https://help.osf.io/article/330‐welcome‐to‐registrations).

There are many ways that research can be designed, analysed, interpreted and communicated, and these are all open to potential misuse (Andrade, 2021; Mela, 2007; Stefan & Schönbrodt, 2023). Conflicts of interest, bias and inappropriate research practices are not limited to industry‐related research, although this may get greater scrutiny (Mela, 2024). A large part of the risk to integrity and replicability in both academic and industry research arises from failure to state and adhere to clear pre‐definition of the primary and other outcomes, aligned to correspondingly appropriate and detailed a priori statistical analysis plans. Many researchers find it daunting to commit to detailed data handling and pre‐planned statistical analysis procedures before starting data collection. Yet, these decisions are clearly better taken earlier than later and can also sharpen thinking about the outcome measures themselves. Moreover, failure to do so leaves the door open to bias and data manipulation practices arising through intent or naivety (Andrade, 2021; Schwab et al., 2022; Stefan & Schönbrodt, 2023). Especially in the case of ‘disappointing’ results, a clear a priori statistical plan safeguards against after‐the‐fact inclinations to re‐define the study intent (‘HARKing’: Hypothesising After the Results are Known) or to continually re‐analyse the data in other ways (‘p‐hacking’). A pre‐defined statistical plan does not preclude undertaking other, results‐led analyses, but these must be clearly framed as posthoc hypotheses.

Plans for any type of research (intervention, observational, systematic review) can and should therefore be recorded in advance in a public registry, and publication of a detailed protocol is also advised. Primary, secondary and exploratory outcomes should be clearly specified a priori, with a detailed data analysis plan for each. For human intervention trials, the per‐protocol and intention‐to‐treat data sets should be defined, as well as protocol deviations, covariates and outlier criteria. In addition to statistical significance, it may be helpful to also consider criteria for practical significance (what would be a meaningful effect size?) and adverse events, to aid objective interpretation of results. Results should be blinded until the research is complete and data locked (= no ‘peeking’ at intermediate analyses or reporting of ‘preliminary’ results). Where possible, data files should also be available for sharing, either from a public repository or on request.

All of these steps and others reinforce research integrity, transparency and replicability, regardless of the nature of research funding or partners. Everyone benefits from this disciplined approach. For commercial collaborations specifically, this also helps assure independence of the academic researchers and provides a strong and objective defence against possible suggestions of (industry) interference or bias. Nevertheless, there should always be a clear disclosure of the funding and role of the academic and industry authors and institutions. This is usually required by journals. Moreover, for complete transparency, it is appropriate to declare any current or prior working relationships with industry in other publications and presentations within the same or related topic areas.

**Table:** 

**Ensuring independence and integrity:**
Discipline, rigour and transparency in methodology and analyses are paramount: agree and publicly register detailed design, data management and statistical plans in advance.
Never suggest your research is intended to *prove* something. It is designed to *test* something. Acknowledge the risks.
Consider preparing ‘outcome scenarios’ in advance, describing and agreeing how different possible results would be interpreted.

## RECOGNISING WHEN THINGS ARE GOING WRONG

Serious issues in research collaborations can usually be traced to naivety or misunderstandings amongst the partners, on either the academic or industry side. The best way to address these is early and head‐on. Do not let the concerns fester: make personal contact and talk through them honestly. Unfortunately, there can also be more fundamental concerns related to principles of trust and research integrity.

Most companies, especially those with a broad portfolio and experience in academic collaborations, have high standards for ethics and integrity in research. Their R&D scientists and nutritionists understand the inherent risks in research and should appreciate the value of getting reliable and decisive results—even if they are not the ‘desired’ direction. But there are also individuals and organisations with lower standards and a different corporate culture, and perhaps more limited scientific expertise in nutrition and health research.

For academics working with or considering a position in industry, it is valuable to observe and perhaps probe the role of science experts in companies and how they fit within the wider corporate structure. In the best case, there is a close working relationship but also a clear distinction between science and marketing. Science experts are relied upon to honestly communicate research evidence and emerging scientific consensus within companies, and identify where this may present risks and opportunities for the portfolio. They are expected to communicate where challenges arise and propose alternatives to address them. Marketing is ultimately responsible for how that information is used (or not!) to influence the direction of innovation and communication.

Issues of scientific integrity within commercial organisations are most likely to arise when there is a lack of dedicated corporate ethical oversight, and distinctions between science‐based evidence and marketing interests become blurred. The nutrition function may be subordinate to marketing, and technical experts incentivised to deliver ‘good news’ while playing down risks, thus to tell senior managers what they want to hear, rather than sometimes difficult truths. At the extreme, there are industry (but also academic) scientists who lose their objectivity and become complicit in ‘defending the indefensible’, engaging in questionable research practices, intentionally distorting evidence or applying degrees of pressure on collaborators to do so as well. This can be intimidating for ECRs, who may lack the experience to recognise the border between acceptable and unacceptable practices, or the confidence to confront others. This further underscores the need to understand the principles of research integrity and ensure these are transparently and rigorously implemented at all stages of the research process. Not only is that the right thing to do, but it also offers a valuable recourse if issues arise later.

**Table:** 

**Industry nutrition warning signs**
Corporate culture:
Evangelical belief in products.
Nutrition subordinate to marketing.
Twist, deflect from the evidence.
Defend the indefensible.
Internal science experts:
See their role as defending products.
Lack objectivity.
Selectively (mis)use scientific evidence.

## CONCLUSIONS

Working with industry can be rewarding and enlightening for ECRs. It offers opportunities to address relevant scientific questions, learn how research gets from the laboratory to the marketplace and develop professional relationships and knowledge outside of the usual academic circles. In most cases, academic and industry scientists share similar interests and values, and the collaborations go fairly smoothly. Nevertheless, academics need to recognise and understand differences in the nature of specific companies and the industry environment, and that these can sometimes present challenges. Table 3 attempts to briefly summarise some of the key ‘Do/Do not’ messages for developing and managing a successful academic‐industry working collaboration. Most importantly, all parties benefit when research is well‐designed and carried out with the highest quality and integrity.

**TABLE 3:** Summary ‘Do’ and ‘Do not’ guidance for ECRs approaching and working with industry.

Do…	
✓ Know the companies, their portfolio and challenges	
✓ Try to find/approach the right contacts (usually R&D, not marketing)	
✓ Remember: It's a business, not a charity or research council	
✓ Think realistically: Focus on tangible deliverables from the research/collaboration (What do they get? How could this eventually work or look? Who will benefit?)	
✓ Emphasise the ‘need to do’ (addressing business and public health priorities), not ‘nice to know’ (curiosity, academic interest)	
✓ Promise quality, not a specific (positive) result	
✓ Be clear about the industry commitment (e.g. letter of support, cash/in‐kind, etc.)	
✓ Understand contract basics (commitments, costs, publication, IP)	
✓ Keep to the agreed project plan, deadlines and reporting schedules	
✓ Pro‐actively update partners, especially any problems, changes, delays	
✓ Be neutral and honest about results. ‘Negative’ results from good research should not affect future opportunities for collaboration	

Do not…	
✗ Say or write more than is needed to get your basic idea across	
✗ Approach with urgent requests	
✗ Presume that products are free/easy/quick to arrange (especially novel products or prototypes). They usually are NOT	
✗ Assume companies have large, uncommitted research budgets	
✗ Confuse research collaboration with endorsement for a company or products	
✗ Keep silent if you have concerns about scientific quality, ethics or integrity	
✗ Try to tell companies how they should run their business (!)	

## CONFLICT OF INTEREST STATEMENT

The views expressed here are solely those of the author, and no other individual or organisation had any role in the conception or initial drafting of this manuscript. The author is an advisor to a number of academic research projects, and a member of the UK Scientific Advisory Committee on Nutrition, which maintains a full list of his declared interests. He is a shareholder and was until 2019 an employee in Unilever, and has since provided independent scientific consultancy to Unilever, Cargill Inc., Danone, CBC Israel and Tate & Lyle PLC.

## Data Availability

Data sharing is not applicable to this article as no new data were created or analyzed in this study.
